# "The Hospital" Nursing Mirror

**Published:** 1901-07-06

**Authors:** 


### "The Hospital" Nursing Mirror.

Being the Nursing Section of "The Hospital."

[Contributions for this Section of "The Hospital" should be addressed to the Editor, "The Hospital" Nursing Mirror, 28 & 29 Southampton Street Strand, London, W.C.]

### Notes on News from the Nursing World.

### THE ROYAL RECEPTION.

The nursing event of the week is fully described in another page. Although necessarily not a public function, the reception by Queen Alexandra of the Queen Victoria Jubilee Nurses was in all respects a notable ceremony. It was one more evidence of the deep and abiding interest which Her Majesty feels in the development of the nursing movement; it afforded a remarkable demonstration of the growth of the useful and important organisation which owes its existence to the late beloved Sovereign; and it was a red-letter day in the lives of every nurse who took part in it. Some had been guests at Marlborough House before. As our readers know so well, Queen Alexandra, as Princess of Wales, has on four occasions welcomed the nurses of the Royal National Pension Fund in their thousands, just as in her capacity of Queen Consort, she has now welcomed the Jubilee Nurses in their hundreds; and among the many who, in response to Her Majesty's invitation, temporarily relinquished their duties to the sick and suffering in various parts of the country in order to partake of her gracious hospitality were a number who had already enjoyed the privilege. We may be sure that there was not one present at the Royal reception, whether for the first time or otherwise, who did not thoroughly appreciate the distinction of being singled out by the most illustrious lady in the land for recognition and honour—a distinction all the more to be prized because it was meant, before all things, as a tribute to the character of the work in which the "Queen's" Nurses are engaged.

### ROYAL NATIONAL PENSION FUND FOR NURSES.

We are requested by the secretary of the Pension Fund to ask nurses who are entitled to receive a certificate from H.M. Queen Alexandra, but who have not been present at a reception, though still members of the Fund, to send their name and address, with their policy number, on a postcard to the Secretary, Royal National Pension Fund for Nurses, 28 Finsbury Pavement, London, E.C. Nurses are asked to use postcards, not letters; to begin their postcard with the word "Certificate" in the left-hand top corner; and not to refer to any other matter on the postcard.

### ARMY SISTERS.

An interesting letter from an Army nursing sister who is now serving in South Africa appears in another column. The writer, it will be observed, directly challenges the statement of a member of the Army Nursing Reserve, who, in our issue of April 20th, said that the regular Army sister is expected to do very little in the wards "beyond giving the medicines and stimulants, going round with the doctors, and charting the temperatures." According to "A. N. S.," instead of the duties of an Army sister under the existing regulations being less onerous and varied than those of a sister in a civil hospital, they are "far more" so; and she proceeds, at any rate, to show that they are not so limited as the author of the article she criticises indicated. Two points in her letter merit the attention of nurses in civil hospitals whose knowledge of nursing in the Army is confined to the period of the present war. Thus, she remarks: "My experience in the Army has been that a sister's patients are ever present with her, whilst in civil hospitals her patients can be handed over to a competent nurse and then the strain is relaxed for a time at least." She also observes that "nurses who have never been in the Army before the war started in 1899 can in no way be judges of the nursing in the same Army in times of peace." This is quite true. But we are sure that the Army Reserve sister who supplied us with such an admirable account of the work at Deelfontein had not the least idea of attacking the members of the Army Nursing Service, and, indeed, her comments cannot reasonably be asserted to have been in the slightest way derogatory to them.

### A SUGGESTION TO MR. BRODRICK.

The Secretary of State for War is himself the chairman of the committee which he has appointed to consider a scheme that he has drawn up for the future organisation of the Army Medical Service. The members of the committee are all men, but this need not prevent them from ascertaining the views of women who are not less experts than themselves. The nursing service is an increasingly important branch of the Army Medical Department, and before any scheme of reform is finally approved, an opportunity should be taken to learn the opinions both of matrons of experience and also of Army sisters who are familiar with the details of the existing system, and know where it might be improved.

### THE OPENING IN BRITISH COLUMBIA.

Our intimation, a fortnight ago, that half a score of nurses were wanted in British Columbia has brought us a very large number of letters from nurses anxious to profit by the opening. The whole of them have been forwarded to our correspondent at Victoria, and as soon as we receive a reply from her it will be published in our columns for the benefit of all who are interested in the matter. Meanwhile, it may be interesting to some of the applicants to learn that Victoria, which 40 years ago contained only 3,500 whites, has now about 25,000, and is the wealthiest city on the Pacific Coast. It is also extremely healthy. The Provincial Royal Jubilee Hospital was erected in 1890 in commemoration of Queen Victoria's Jubilee, and is open for the reception of paying patients as well as for that of the poor of the city, who are, of course, treated free. Victoria is the seat of the Provincial Government and the home of the Lieutenant-Governor. Electric cars were introduced long ago, and the advantages which the city offers for residential purposes are very considerable.

### A THANKOFFERING TO NURSES IN RHODESIA.

A number of members of the Imperial Yeomanry have just despatched from London to the Victoria Hospital, Rhodesia, a handsome upright grand piano. It is intended as a thankoffering to the nurses who ministered to them when they were sick and wounded, and will doubtless be greatly appreciated by the recipients.

### NURSING IN WEI-HAI-WEI.

A NURSE, who has lately returned from China, supplies us with some particulars about her work in Wei-Hai-Wei. She started last August with another trained nurse, I. R. N. canteen having been converted into a hospital. Inside the main entrance was a courtyard round which were the games-room, servers' quarters, and some outbuildings; up a flight of steps was the dining-room, which formed one side of the quadrangle; the general bar, billiard room, and manager's quarters filled the other sides respectively; and behind was the skittle alley, used as a baggage room, and a couple of big empty rooms. The nurses found awaiting their attention fifty-five patients, both medical and surgical, from Admiral Seymour's first relief column. It took them some little time to get the place ready for straightforward work, as there had been no regular staff. They had six orderlies, untrained men. A Chinese compradore catered for the hospital. In spite of numerous difficulties, the patients managed to progress, and in the beginning of September a draught of thirty was sent home. This left twenty cases remaining, but in the course of a week every bed was filled, many of the sick coming from Pekin. It was then that the sick work really began in Wei-Hai-Wei. The new cases were chiefly typhoid and dysentery, and the epidemic was very severe while it lasted, which was until the beginning of November. With the cold weather the number of new cases lessened, and the hospital was closed at the end of November, the remaining patients being transferred to the Club Hospital. During the epidemic the staff was increased by three nurses, but with frequent sickness also amongst these there were seldom six on duty at the same time.

### THE TUBERCULOSIS CONGRESS.

THE nurses who attend the British Congress on Tuberculosis, to be held in the Queen's Hall, London, from the 22nd to the 26th of this month, will probably find many points in the nursing of the disease elucidated by the addresses and the discussions, which may be of use to them in the future. But, of course, the number present will necessarily be very limited, so that we intend to give a *résumé* of any details which may be likely to prove of assistance to nurses, not only in fighting phthisis itself, but in preventing as much as possible the spread of it amongst the poor. The nurses in the hospitals and the sanatoria for consumption naturally run only a trifling risk of infection, because they know how to take proper precautions and have the needful appliances at hand. But it is to the district nurse that the hardest part of the battle falls, because she often has to attend a patient whose death she feels she can only defer, and yet all through the weary months she must strive to prevent the disease being passed on to friends and relatives. A case has lately come under our notice of a young servant leaving her situation to nurse a cousin suffering from consumption. The cousin died, and the next year the ex-housemaid "nursed" the brother of her first patient. He died also, and her attention was turned to a sister of her own who had helped in the amateur nursing. Before the third year was out she had buried her sister. Now she herself is threatened with the disease. If there had been a trained nurse in the country village to show her the danger of infection, and how to guard against it, she being a fairly intelligent girl, the chances are that some of the lives might have been spared. District nurses can, perhaps, by their teaching, do more to stop the progress of phthisis in this country than even the medical men themselves.

### THE AGE QUESTION AT LIVERPOOL WORKHOUSE INFIRMARY.

THE Liverpool Workhouse Committee have decided not to make any alteration in the minimum age at which probationers are admitted. For some years the minimum has been 23, but it was proposed that this should be reduced. Twenty-three, however, is quite early enough for a girl to enter upon a nursing career. In many instances it has been considered advisable to make the minimum 25; but it is only reasonable that with the lengthening of the training the age at which it should be commenced should be lowered. The regulation in force at the Liverpool Workhouse Infirmary seems to have worked well and cannot possibly be described as harsh.

### CORK AND LILLE.

WHILE the trustees of a Cork hospital are putting back the clock, we notice that in France a laudable attempt is to be made to keep pace with the modern developments of nursing. The Lille Town Council have decided to replace the members of a religious society who have been responsible for the nursing at the local hospitals by lay nurses. The reason cited for the change is that a member of the council, who is also a doctor, declared that the hospitals were neglected, "owing to so much time being spent upon religious observances." We do not, of course, know how far this assertion was founded upon fact, but as it carried conviction to the entire council we conclude that the medical man in question was able to give chapter and verse in support of his statement. At all events, the decision to place the nursing at the Lille hospitals in the hands of trained lay nurses is a step in the right direction.

### THE NURSES OF PRESTON ROYAL INFIRMARY.

LAST Wednesday afternoon prizes were awarded to the nurses of the Preston and County of Lancaster Queen Victoria Royal Infirmary, the result of the examinations recently held after the annual course of lectures on physiology, anatomy, and general nursing given by the senior house-surgeon and the matron. The prize-winners were Nurses Madeline Winter, Hannah Nealy, and Bessie Mitchell. The Chairman, who gave the prizes, in presenting them, made a suitable speech encouraging the nurses by highly commending their work during the past year. The nurses were entertained to tea in the garden by the matron. Tennis and ping-pong were provided for their amusement. At the close of the proceedings Miss B. A. Cookes, the assistant-matron, who is leaving to take up her new appointment of matron of the St. Mary's Hospital, Plaistow, was presented by the nursing staff with a pair of pictures.

### A BOGUS NURSE AT TEIGNMOUTH.

WE are glad to hear that a bogus nurse has been laid by the heels at Teignmouth. Annie Wolfe, who described herself as "from Guy's Hospital, London," arrived at Teignmouth on June 18th and established herself in lodgings in Bitton Street. Then she represented that three friends would take the rest of the rooms. On various pretences she managed to remain for a fortnight, and at last she gave out that she was going to relieve a nurse who was attending the child of a local medico. This led to her undoing, for it was proved that no nurse was there and no child ill. The police were put on her track, and when they arrested her she was found to be penniless. In the police cells she frankly confessed to her deception and gave two addresses in London, but nothing was known of her at either. The magistrates provided her with a permanent address at Exeter Gaol for a month, where she will experience the treatment given to "A" class of offenders. Perhaps she will have learnt a lesson by the time she emerges, and will cease masquerading under false colours. People who attempt in this manner to bring an honourable profession into disrepute should be severely dealt with, for such frauds have the effect of bringing an undeserved stigma on nurses in general.

### REVISION OF SALARIES BY THE METROPOLITAN ASYLUMS BOARD.

At a meeting of the Metropolitan Asylums Board on Saturday the Hospitals Committee reported that they had received petitions from the assistant matrons, housekeepers, and superintendents of night nurses asking for the revision of their salaries. Under the existing scale the salaries and emoluments are:—Assistant matrons, £50 per annum, with board, lodging, and washing; housekeepers, £50 per annum, with board, lodging, and washing; superintendents of night nurses, £42 per annum, rising £2 to £46, with board, lodging, and washing. The committee stated that they had carefully considered these representations and had ascertained the salaries paid to persons occupying similar positions in certain of the general hospitals and poor law infirmaries. They thought that a case had been established by the assistant matrons, and they recommended that the salary of trained assistant matrons in hospitals service be increased from £50 to £60 per annum, and that uniform be added to their existing emoluments. With regard to the housekeepers, the committee could not advise that their salary should be increased, but they thought that the emolument of uniform should be added to their existing emoluments and they recommended accordingly. The articles of uniform of assistant matrons and housekeepers should, they thought, be similar to those allowed to superintendents of night nurses with the exception of material for dress of the assistant matrons, which should be different for the sake of discipline. They therefore recommended that in the event of the board adopting the foregoing recommendations it be referred to the clerk to make the necessary alterations in the wages and uniform scales for infectious hospitals in respect of the salary and emoluments of assistant matrons, and the emoluments of housekeepers in the hospital service of the board. As to the superintendents of night nurses, the committee stated that they were not prepared to submit any recommendation to the board at the present time. The report was adopted, and notice given that the recommendations which it contained would be formally moved at the next meeting of the board.

### A SUCCESSFUL SALE OF WORK.

It is doubtful whether the value of sales of work as a means of increasing the funds of nursing associations are sufficiently recognised. The ladies' committee of Peckham Nursing Association have just shown what can be effected in this way by good management and enthusiasm. A sale of work which was held for two days last month in the interests of the organisation has yielded, after payment of all expenses, the substantial sum of £40. There are also left on hand numerous useful articles, which it is proposed to offer for sale in December. The fact is that many people who cannot give money are often able to contribute work, while others are willing to supplement modest pecuniary assistance by making articles which, while not costing much except in time, find ready purchasers, if the wise policy of charging moderate prices is strictly adhered to.

### MADE IN GERMANY.

A description has been given of "A Municipal Nursing Home" at Dusseldorf, on the Rhine. But the title is misleading. The so-called nursing home is really a municipal refuge for single persons of both sexes who are more or less unable to support themselves on account of age, bad health, or other causes. It is, however, administered by a matron and a number of sisters belonging to the Franciscan Order. They are not paid salaries, but receive £90 a year among them for their clothing. We do not know if there is much active nursing to be done, but the maintenance of the "Nursing Home" costs the ratepayers of Dusseldorf between £7,000 and £8,000 a year.

### HEREFORD NURSING HOME.

Considerable financial support has been already given to the proposed "Victoria Memorial" Nursing Home at Hereford. The building is to afford accommodation for the private nursing staff, which has been managed by the authorities of the Herefordshire General Hospital for the last five years, and altogether there will be provision for a minimum of twelve nurses. It is intended to receive two patients under the care of their own medical men, but the nursing of the poor in the city and county will, of course, be the main object in view. A sum exceeding £2,000 will, it is estimated, be required for the completion of the home and the necessary appliances.

### SHORT ITEMS.

The Queen has consented to continue the patronage of Her late Majesty Queen Victoria to the Up-country Nursing Association for Europeans in India.—Meetings have been held this week to organise committees in Westminster and Marylebone on behalf of the Women's Memorial to Queen Victoria in connection with the "Queen's" Nurses.—Mr. John Martineau has just sent a donation of £500 to the Women's Memorial to Queen Victoria.—A successful bazaar in aid of the funds of the Oxygen Hospital, 2 Fitzroy Square, was held last Wednesday and Thursday at the residence of Lady Nottage, Collingham Road, South Kensington. The Baroness Burdett-Coutts performed the opening ceremony the first day, and the Marchioness of Granby the second.—Nursing Sister Alice Grace Rees has been discharged from hospital to duty in South Africa.—The following nurses arrived from South Africa on Saturday on board the s.s. *Simla*:—Sister M. Mark (A.N.S.), Sisters B. A. Roberts, H. N. O'Connor, M. E. Ireland, F. J. E. Smith, and M. O'Shanahan. Sisters O'Connor, Ireland, and Smith appear in the official roll as time-expired; the others rejoin the ship. Sisters E. Ward, C. Walker, and S. C. Cheetham are invalided home. Sisters A. D. Hook, E. L. R. Ferguson, D. B. Hyland, H. T. Henderson, and S. B. Peers arrived in the *Orotava* on Tuesday.—In consequence of the appalling heat in New York this week, the Roosevelt Hospital has been so crowded with heat-prostration patients that on Tuesday the nurses played the hose on eight patients lying on the lawn instead of applying the usual ice-packing treatment.—Last week the St. Olave's Guardians agreed, subject to the approval of the Local Government Board, to give £100 towards the cost of a Nurses' Home to be erected by the St. Olave's Nursing Association.

### Lectures to Nurses on Medicine and Medical Nursing.

By NORMAN DALTON, M.D. London, F.R.C.P., Physician to King's College Hospital.

### LECTURE XIV.—DISEASES OF THE ORGANS OF RESPIRATION.

### DYSPNŒA—ASTHMA.

THERE are certain symptoms which occur in nearly all the diseases of the respiratory organs and these must be first considered.

*Shortness of Breath* (dyspnœa).—In all cases of dyspnœa, nurses must count the number of respirations per minute. They must remember, however, that there may be great distress of breathing without any increase in the number of respirations; for instance in asthma, and in diphtheria (when the larynx is obstructed), the patient cannot breathe rapidly because each breath takes such a long time to draw. He would like to breathe quickly but can only breathe forcibly. The breaths should be counted without attracting the patient's attention, as he may instinctively alter the rate without wishing to do so; but in urgent dyspnœa this precaution may be omitted. Normally about 16 breaths are taken per minute, but in children the rate is quicker.

Dyspnœa is always most severe when the disease is one which has come on suddenly, such as diphtheria, which may rapidly cause the windpipe to be filled up with membrane; or pneumonia, which may rapidly incapacitate one-half of a lung. The reason is that the patient is usually in good health when the disease sets in and is consuming a large amount of oxygen. Hence his tissues require a great deal of oxygen and they feel the sudden diminution in the supply very acutely. On the other hand, in consumption, the lung tisssue is destroyed slowly (except in a few acute cases), and the body wastes at the same time. Hence there is less tissue to use up the oxygen and the diminution in the supply causes less distress.

There are different types of breathing which a nurse should be able to recognise. In "acute pneumonia" the respirations are very rapid, perhaps sixty a minute, the alænasi work and the face is flushed. The movement of the chest is fairly free. In "capillary bronchitis" and "broncho-pneumonia" in children the dyspnœa is of the same type, but often the lower part of the chest does not expand freely during inspiration. In "laryngeal obstruction," such as may occur in acute laryngitis, in diphtheria, and in cancer of the larynx, the respirations are as quick as the patient can manage (see above). They are very forcible, and at each inspiration the neck above the sternum, and in children the lower ribs, are sucked inwards. There is also a loud whistling or crowing sound called "stridor" with the inspiration, and sometimes with the expiration also. The noise is produced by the forcible drawing of the air through the narrowed larynx. This variety is called "stridulous" breathing, and it is of vital importance for a nurse to be able to recognise it at once, for it may come on quite suddenly in a case of diphtheria, or more rarely in a case of measles. In both cases it indicates that the larynx is diseased and that urgent treatment (in the case of diphtheria, tracheotomy) is called for. In the case of measles, a nurse may on her own responsibility set the steam kettle going and arrange it so that the steam must be inhaled by the patient. A tent may also be erected over the bed so as to concentrate the vapour. "Tracheal" breathing, which occurs when an aneurism compresses the trachea, has much the same character as laryngeal breathing, but there is less stridor.

In "asthma" the patient breathes very forcibly, using his voluntary muscles of respiration, and often holding on to some firm object in order to aid the action of those muscles. Inspiration is short and ineffectual, while expiration is long drawn out and accompanied by a loud wheezing sound. Asthma is probably caused by a spasmodic contraction of the muscular fibres in the walls of the bronchial tubes. This contraction narrows the tubes and makes it difficult for the air to get in and out of the lungs. Some pathologists, however, think that it is due to a peculiar kind of inflammation of the bronchi. It may occur quite suddenly in a healthy person, but most often nurses see it in patients with chronic bronchitis. In all cases it is especially liable to come on in the early morning, at about 3 or 4 A.M.; hence a nurse should be able to recognise it, as she will probably be alone with her patient. Asthma is very distressing to the patient, very painful to witness, and very alarming to the friends. But unless the patient is very ill with bronchitis, asthma is not dangerous in itself, so that the nurse can reassure the friends. It would also be safe for her to administer an extra dose of brandy and a small cup of strong black coffee, provided that stimulants have not, for any reason, been forbidden in the particular case. In many cases the attack of asthma is brought on by something irritating the stomach, so that asthmatics should always take small quantities of nourishment at a time, particularly in the evening, and when the attack is on. This rule, by the way, applies to all cases of dyspnœa, namely, that the meals or feeds must be small in quantity because a full stomach presses against the diaphragm and impedes its action.

In some cases of kidney disease there is a peculiar kind of dyspnœa which is called "renal asthma." The breathing may be wheezing as in ordinary asthma, but often it is merely rapid and there is nothing to be heard even with the stethoscope. It causes great distress to the patient and is often accompanied by a short cough without expectoration. It is due to a poison in the blood.

Many cases of diabetes terminate in a state of unconsciousness or "coma" which comes on in the course of a few hours. During this coma the breathing becomes very rapid but it is very free; *i.e.*, although the breaths follow each other in rapid succession, each one expands the chest freely and the air enters freely into all parts of the lungs. The breathing is noisy from the rapid inrush and outrush of the air, but there is no stridor or wheezing, and nothing to be heard with the stethoscope. The trouble is not that the air has any difficulty in reaching the blood in the lungs, but that the blood is poisoned and cannot take up the oxygen.

It must be remembered that, if there be pain on drawing a deep breath, the patient takes care to make his breaths shallow, and, consequently, is obliged to breathe more quickly in order to get the right amount of oxygen in a given time. This is another kind of dyspnœa, and it occurs in pleurisy, in intercostal rheumatism, in peritonitis, &c. In pleurisy, one side of the chest expands more than the other at each inspiration, because the pain is only on one side. In peritonitis, the pain prevents the patient from using his diaphragm, so that breathing has to be carried on by the thoracic muscles only. In such cases, therefore, the upper part of the chest alone expands during inspiration, and the breathing is called "thoracic." On the other hand, if the chest walls become very rigid, as sometimes occurs in elderly people, especially when they are emphysematous, the thoracic muscles cannot expand them properly, and thus nearly all the breathing is carried on by the diaphragm, and we can see the abdomen pushed out by the descent of the diaphragm at each inspiration, and fall back during each expiration. This is called "abdominal" breathing. If the thoracic muscles become paralysed, as sometimes occurs in spinal disease or in fracture of the spine, the diaphragm may have to do all the work of respiration unaided, and then the movements of the abdomen are very great. This is called "diaphragmatic" breathing.

In all cases of "dyspnœa" in which there is any chance of recovery such as pneumonia and bronchitis, the rules for nursing are the same as those laid down in the lecture on the nursing of mitral disease.

### Reception of "Queen's" Nurses by Queen Alexandra at Marlborough House.

BY OUR OWN CORRESPONDENTS.

ABOUT one o'clock on Wednesday afternoon, in brilliant sunshine, groups of blue-uniformed nurses were seen making their way, on foot or in cabs, to the Scottish Drill Hall, in Buckingham Street; indeed, one seemed to see them wherever one looked.

### DRILLING THE NURSES.

Inside the hall, under the generalship of Mr. Sydney Holland and Miss Peter, the nurses were being drilled into their curtseys, ready for the ceremony at Marlborough House. How well they learnt their lesson was evident a couple of hours later, when they filed past the Royal group; and it is to be hoped that their instructors felt rewarded for the pains they had bestowed on this important point. Mr. Holland himself personated Majesty, standing in a conspicuous position, while the nurses were drawn up in an open square. "Now you nurses," he said, "must learn to be very quick and smart, and do everything I tell you." So the nurses obediently arranged themselves, four deep, round the hall, [the little ones in front, and as if there could be any doubt as to relative heights, they were told to look into one another's eyes. Then the order in which the recipients of badges, brassards, and certificates were to come, was arranged, with a view to as little loss of time as possible. This having been done, the nurses practised the filing past.

### THE PROCESSION THROUGH THE PARK.

Shortly before two o'clock, the expectant crowds waiting in the Park were rewarded; the blue and white procession—the nurses wearing aprons and carrying their cloaks on their arms—came into sight, and by about a quarter past two, those waiting outside the Friary Gate of Marlborough House saw them arrive, led by the Master of St. Katharine's, Mr. Sidney Holland, and Mr. Harold Boulton. The large gates were opened, the procession passed in, and marched in orderly fashion along the gravel walk, skirting the velvet lawn with its gay flower-bed in the centre, to their places, forming a hollow square of which the inner side faced Marlborough House. The great trees spread a delicious shade over parts of the assembly, but on others the sun's rays descended with summer intensity, and umbrellas quickly went up. A pretty kaleidoscope effect of colour was produced when those standing on the path parallel with the front of the house were asked to climb the bank, so as to make room for another row, one saw a flash of white crosses of apron-strings on blue galatea, as they obeyed the order. Next the band of the Grenadier Guards arrived, and then for about half an hour the photographers were hard at work. The umbrellas went down for the purpose, and the officials of the Council stopped half-way across the lawn so as to be included in the view.

The specially invited guests were as follows:—The President of the Council of the Institute (the Rev. the Master of St. Katharine's), H.R.H. Princess Louise, President of the Scottish Council, the Earl of Derby, K.G., Lord Rowton, K.C.V.O., C.B., Sir Fleetwood Edwards, K.C.B., trustees; the Duke of Portland, K.G., the Earl of Meath, Lord Balcarres, M.P., Lord Alwyne Compton, M.P., the Hon. Sydney Holland (hon. treasurer Queen's Nurses' Endowment Fund), Sir Dyce Duckworth, M.D., Major-General Sir Stanley Clarke, K.C.V.O., C.M.G., Mr. R. D. B. Acland, Mr. Henry Bonham Carter, Mr. Thomas Bryant, F.R.C.S., Rev. Dacre Craven, Mr. R. O. B. Furlong, C.B., Mr. Howard Horley (hon. treasurer Queen's Nurses' Endowment Fund), Mr. L. A. Teeling, Mr. A. Williamson, the Countess of Lonsdale, the Countess of Selborne, Lady Lucy Hicks-Beach, Lady Penrhyn, Hon. Lady Ponsonby, Amy Lady Tate, Miss Guthrie Wright, (hon. secretary Scottish Branch), Miss Olga Hertz, Mrs. Power Lalor, Mrs. Wm. Minet, Miss Rosalind Paget, Mrs. Acland, Mr. W. G. Rathbone (hon. secretary of the Institute), Mr. Harold Boulton (hon. treasurer of Institute, and hon. secretary Queen's Nurses' Endowment Fund, and Women's Memorial Fund), Members of the Council, and the Duchess of Portland, and the Marquis of Dufferin and Ava.

### THE CEREMONY.

A few minutes after three o'clock had struck, the Queen descended the steps and stood under the canopy. This was the shimiana, or tent, used at the presentation of war medals on the Horse Guards Parade not long ago. The King accompanied the Queen, and H.R.H. Princess Louise, Duchess of Argyll, who is president of the Scottish branch, was also present. On the top step were the little Princess Victoria and Prince George of York, the Princess in a white frock, and the Prince in a white and blue sailor suit. When the Royal party appeared, the band struck up the National Anthem, and those nurses who had chairs rose. Mr. Sydney Holland presented the Master of St Katharine's to Her Majesty, who stood, of course dressed in deep mourning, her only ornaments being a pearl brooch and dress chain. Miss Peter was next presented, and then the Master of St. Katharine's read the following address:—

### THE ADDRESS.

*May it please Your Majesty*—

On this occasion when Your Majesty is graciously pleased to present the Badges and Certificates to the Queen's Nurses it may be desirable to state briefly the conditions under which these are given:—

The Badges which are to be distributed to the nurses who are entitled to hold them are of two kinds:

First, the Silver Badge, which is given to the Superintendent of a Nursing Home, and is the mark of the Office which she holds. The badge is returned when she ceases to be a Superintendent and is replaced by a small Silver Badge which she is entitled to keep if she has completed not less than six years' satisfactory service dating from her appointment as a Queen's Nurse.

Secondly, the Bronze Badge, which is given to the nurse when her name is placed on the "Roll" after she has fulfilled the conditions as to hospital and district training. The badge may only be worn so long as the nurse works under an association affiliated to the Institute; when she ceases to do this it must be returned. But when a nurse has been working as a Queen's Nurse for not less than six years from the date of her appointment and has reason to give up her work, she is granted a "Leaving Badge," which she may retain as a mark of having done service under the Institute. There are five such badges to be presented to-day.

Besides the badges there are certificates to be presented to two classes of nurses:—

(1) To those who, having been trained at the expense of the Institute, have signed an agreement to work wherever the Council may require their services and have completed not less than two years' satisfactory service.

There are fifty-three certificates of this class to be presented.

(2) To Queen's Nurses who have completed four years' work to the satisfaction of the Institute, the difference between these and the former class of certificates being that the holders of them were employed in district work by associations who trained their own nurses according to the provisions laid down by the Institute, but were not under any agreement with the Institute. There are 11 certificates of this class to be presented.

As regards the general position as to number of nursing associations in the United Kingdom affiliated to the Institute, there are in

| | |
|---|---|
| England ... ... ... | 247 |
| Wales ... ... ... | 65 |
| Scotland ... ... ... | 139 |
| Ireland ... ... ... | 55 |
| Total ... ... | 506 |

The number of nurses on the Queen's Roll at the present date is about 960.

There are several who have been in the service of the Institute since its foundation in 1889.

Of the 960 whose names are now upon the roll, 198 are to receive badges at your Majesty's hands.

The supervision of the nursing work is undertaken by
The General Superintendent of the Institute, and

*In England, Wales, and Ireland* by
7 Inspectors,
58 Superintendents of Nursing Homes.

*In Scotland* by
The Superintendent of the Scottish Branch
2 Inspectors
5 Superintendents of Nursing Homes.

The rapid increase of the work of nursing the sick poor in their own homes and the general interest that is taken in it, is a standing witness to the lasting value of the work which is associated with the name of Her late Majesty Queen Victoria. It is of the very rarest occurrence that nursing associations once formed are given up and cease to work because the people are indifferent about the services of a nurse who is resident among them. The thought and opinion expressed far and wide is that of the greatest gratitude for the self-denying and skilful work which the nurses are carrying on wherever they are appointed. Testimony is over and over again given of the appreciation by the poor of the nurses' work and of the good they are doing. It is not infrequently reported that the name of the Queen's Nurse is a household word in a town, and that she has endeared herself to all classes of the community. There is a universal acknowledgment of the unflinching bravery with which they deal with all kinds of terrible sickness and disease and of the tenderness and sympathy which makes their presence in the chambers of sickness and suffering welcome and trusted. It has also been reported to us that a Sanitary Inspector has said that he could tell the houses where the Queen's Nurses had been carrying out their mission.

The Council cannot but hope that as the knowledge of the work of the Institute, which has become a National Work, is more widely known, it will command that practical sympathy which will enable it to continue and extend its efforts in perfecting the work which has been entrusted to their care. We feel, and we venture to express the conviction, that our hands have been strengthened, and that the devotion of the Queen's Nurses to their work has been kindled afresh by your Majesty's gracious kindness in receiving us here to-day. And we beg to assure Your Majesty as Patron of the Institute that it will be a cause of pride to us, and that we shall make it our highest endeavour to increase the usefulness of a work with which Your Majesty's name is so closely associated and which is such an incalculable blessing to the Sick Poor for whose benefit it was established.

### The Queen's Reply.

The Queen replied as follows:—

"It gives me great pleasure to receive you all here to-day, and it is most gratifying to me to be able to carry on the work founded by our dearly beloved and never-to-be-forgotten Queen Victoria. I have always taken the most sincere interest in nurses and nursing, and it affords me heartfelt satisfaction to be associated with you in your labour of love and charity. I can, indeed, imagine no better or holier calling than that in which you are engaged, of tending the poor and suffering in their own homes in the hour of their greatest need. I shall follow with interest the reports of the institution, and shall anxiously note the progress which you are making from year to year. I pray that God's blessing may rest upon your devoted and unselfish work."

The Queen read her reply, in clear and emphatic tones, from a black-bordered type-written paper, which she afterwards handed to the Master of St. Katharine's. It was signed in her own hand, *Alexandra.*

### Presentation of Badges.

The nurses were then marshalled in the order in which they were to receive the badges, brassards, and certificates; these were arranged on a table, and handed to the Queen by Mr. Sydney Holland, while the names were read by the Master of St. Katharine's.

The following are details of the numbers of nurses who received badges and certificates:—

(*a*) Badges on appointment as Queen's Nurses:—

| | |
|---|---|
| England ... ... ... ... | 117 |
| Scotland ... ... ... ... | 29 |
| Ireland ... ... ... ... | 14 |
| Wales ... ... ... ... | 15 |
| Total ... ... ... | 175 |

(*b*) Badges on leaving the Institute after not less than six years' service as Queen's Nurses:—

| | |
|---|---|
| England ... ... ... ... | 4 |

(*c*) Certificates on the satisfactory completion of certain periods of service:—

| | |
|---|---|
| England ... ... ... ... | 38 |
| Scotland ... ... ... ... | 9 |
| Ireland ... ... ... .. | 2 |
| Wales ... ... ... ... | 6 |
| Total ... ... ... | 55 |
| Total number for Badges and Certificates ... ... ... | 234 |

The Bloomsbury Nurses were the first to receive them, and early in the list came the Lady Hermione Blackwood, daughter of the Marquis and Marchioness of Dufferin and Ava, who has just completed her training in district nursing. Next the certificates were given, and then followed an impressive scene.

### The March Past.

The entire assembly of nurses, 770 in all, filed past the Royal party, each making her curtsey; and, notwithstanding the enormous number, the ceremony, thanks to carefu organisation, was not a long one. Before 4 o'clock it was over, and the proceedings resolved themselves into something of a garden-party type.

### To Nurses.

We invite contributions from any of our readers, and shall be glad to pay for "Notes on News from the Nursing World," or for articles describing nursing experiences, or dealing with any nursing question from an original point of view. The minimum payment for contributions is 5s., but we welcome interesting contributions of a column, or a page, in length. It may be added that notices of entertainments, presentations, and deaths are not paid for, but, of course, we are always glad to receive them. All rejected manuscripts are returned in due course, and all payments for manuscripts used are made as early as possible after the beginning of each quarter.

### Royal British Nurses' Association.

### QUARTERLY COUNCIL MEETING.

DR. GODSON took the chair at the Quarterly General Council Meeting of the R.B.N.A. on Friday. The treasurer's report showed that receipts were £198 15s. 11d.; expenditure, £227 17s. 3d.; bank balance for general purposes, £67 19s. 4d. The Settlement Fund had £450 on deposit account; £81 8s. 9d. in the general account, making a total of £531 8s. 9d. The report was adopted; and the next business was the report of the Executive Committee. This, Mr. Fardon said, was necessarily brief, owing to the short time that had elapsed since the last meeting; it referred chiefly to the annual meeting. He stated that a misapprehension had arisen with regard to the intentions of the Association about their midwives list, appended to the roll of members. It had arisen from the annual report of the Medical Defence Union, published in the *British Medical Journal*. It had been there stated that in future editions of the Midwives' List "a foot-note should be appended to each page that these certificates (of the L.C.S.) were for the training of nurses and not diplomas qualifying for the practice of midwives." It was important that this should at once be contradicted. Such a suggestion was unfair to the members of the Association, whose interests they had to safeguard. They were determined that the members should not suffer in any way on this account. They had agreed to substitute the word "certificate" for the word "diploma," and to make a slight alteration in the heading, and this had been construed into a promise to make a statement that these nurses were not qualified to practise as midwives. This statement had now been authoritatively contradicted, and Mr. Fardon read the whole correspondence relating to the matter. The Association held that nurses who, in addition to their hospital training, had obtained special training in midwifery, were qualified so to act, and they also held that training in a hospital was essential to the proper care of midwifery cases. If any persons were qualified to be midwives, those whose names were on the list were, and the Association intended to continue using the term "midwife," and to make it quite clear that they had no intention of inserting any note to make it appear that such nurses were not qualified. He hoped that the explanation in the forthcoming number of the *Nurses' Journal* (the organ of the Association) would put matters clear.

The CHAIRMAN read a letter from Dr. Cullingworth, suggesting that it was not correct to say that the certificates were for the training of nurses; they were granted to those who had passed an examination in midwifery specially instituted for midwives.

Mr. FARDON said the nurses ought to be congratulated on having such an eminent supporter as Dr. Cullingworth.

The CHAIRMAN said he did not think that by objecting to the word "diploma" there was ever the slightest intention of suggesting that the nurses were not fit to act as midwives, for they were plainly qualified to do so. It depended on how the word was interpreted.

Dr. LESLIE THORNE-THORNE said that if any nurses were qualified to act as midwives, the nurses of the Association were; but he very much doubted if any nurse was qualified to act in every case, *e.g.*, in a case for forceps or craniotomy. In country districts, for instance, when a nurse had been accepted as a midwife, he thought that difficulties might arise in special cases. The word "qualified" seemed to him somewhat misleading. He did not think any nurse with that training only could act in every case, and if she had that training only she ought not to do so.

Mr. FARDON said the word "midwife" had always been used, and always would be used as long as the world lasted, to describe a person who attended women in natural labour. He had never heard of such a person attempting a case which would involve the destruction of the child. He had no doubt at all that this was the general meaning of the term "midwife."

Mrs. LATTER asked whether there would be any alteration in the wording of the list; and Mr. FARDON replied that the Association would not substitute the word "Midwife-nurse," because of the interpretation put upon it by the Medical Defence Union. The report was then seconded and carried without a dissentient vote.

Mr. FARDON said it had been suggested that the Association should adopt a formal resolution on the subject under discussion; he therefore moved that the following be the title and footnote referred to:—*Title*: "List of members who have also obtained certificates of special training as Midwives." *Note*:—"Previous to June, 1895, the certificate granted by the London Obstetrical Society was called by this body a 'diploma.' Since 1895 the word 'certificate' has been substituted, and it should be so understood in the following List."

Miss THOROLD seconded the resolution and it was carried.

The Executive Committee for the ensuing year were then elected, chosen from members of the council. Dr. Wightwick was the only addition to the medical representatives; he was very deeply interested in the Auxiliary Nurses' Society. The following were the ladies proposed to represent the matrons (five vacancies):—Mrs. Latter, Miss Graham (matron Mile End Infirmary), Miss Oldham (matron of Hospital for Epilepsy, Regent's Park), Miss Forrest (superintendent of nurses, Victoria Nursing Home, Bournemouth), Mrs. Redpath (late matron of St. Bartholomew's Hospital). To represent the sisters and nurses:—Miss Grace Gordon, Miss S. Fenwick Hutchinson, Miss Bramwell, Miss Tawney and Miss Janet Bird (representing the private nurses). The proposal being seconded, the vacancies were filled as recommended. Owing to another engagement Dr. Godson's place was then taken by Mr. Alfred Cooper, and the next business was the reading of the bye-laws for the Australian branch. A nurse asked whether, when these had been returned from the Australian branch to receive final adoption by the parent Association, they would be brought before the general members? Mr. Fardon said that it was a matter for the council to deal with, but that any member wishing to look through the bye-laws could do so before they were forwarded to the Australian branch.

### TRAVEL NOTES AND QUERIES.

ACCOMMODATION AT PARAMÉ (Randolph).—The sweet simplicity and modest prices of Paramé are rather things of the past, though living is still fairly reasonable. Unfortunately you are going in the height of the season, when everything is dear. There are three good boarding houses: Miss Waller, Alexandre House; Mrs. Samler, Villa Stella Marie; Miss Colnett, Villa Grazia Maria. Try Miss Waller first; write and ask her terms. A very reasonable hotel is the Hôtel des Baines Pension, from 6 francs. A little more expensive the Hôtel "Quic qu'en Grogne."

THE MOSELLE (Nadar).—At Moselkern, Hôtel Eltz, terms 3½ marks per day; at Carden, Pension Brauer, 3½; at Cochem, Hôtel Kaiser, 3½; at Alf. Pension Nollern, 3½. At Berncastel try the Post. It will be rather dearer than the others. It is impossible to get anything lower than this, and in the height of the season I fear terms will be raised. If they would take you for 70 marks on the Rhine, you know it is really very cheap, only 5s. per day. But I think you will like the Moselle even better. Knokke, in itself ugly, is, of course close to the loveliest mediæval cities, which are full of beauties, but North Belgium has no scenery. The Rhine is certainly very tourist ridden. Let me hear how you get on, and also if you want more help.

TO IRELAND IN SEPTEMBER (Erin).—If you do not go till then you can wait a little for information. I am gathering particulars of tours in Ireland, and will let you know nearer the time. Unfortunately, travelling is still an expensive matter in the Emerald Isle; lack of competition keeps up prices, and one cannot put up in all sorts of out of the way places as in Brittany. The cheap line by the Bristol Navigation Company would be a great help to you as you are so near. Bristol to Dublin, first class return, 15s.; Bristol to Cork, first-class return, 22s. 6d.

### The Compulsory Registration of Midwives.

THE eighth annual general meeting of the Association for the Compulsory Registration of Midwives was held on Friday afternoon, by permission of Lady Balfour of Burleigh, at 47 Cadogan Square. Mr. HEYWOOD JOHNSTONE, M.P., took the chair, and in his opening remarks he said he hoped that this might be the last, or last but one, meeting at which he would have that privilege; a continual dropping wore away stones, but the rock they had to remove was a very hard one, and the process was long. Sir Charles Hall, Recorder of London, had affirmed that in this country anyone could call herself a midwife, and the consequences of this state of affairs had to be faced. Mr. Johnstone quoted a case in which a so-called midwife, in whose hands the patient had died, after having been cautioned by the coroner, had gone straight to another case, which had also proved fatal in her hands. He wanted to point out that details were of minor importance in the Bill providing for the registration of midwives; the two main points were, (1) proper training, and (2) supervision. Last year the second reading of the Bill was assented to in the House of Commons by a large majority, and it went almost unaltered through the Standing Committee on Law; but in spite of the fact that a memorial in its favour was sent to the Leader of the House, signed by medical men, coroners, representatives of hospitals, and others whose special knowledge and experience of the subject entitled their opinion to the utmost consideration, no adequate opportunity was found for its later stages, and the measure therefore lapsed. He believed that opinion on the subject was ripening; and he thought that the Government should be induced to take it up. With a view to getting the public to take an effective interest in the matter, a sub-committee had been formed. Their special function was to bring the question before the working classes, who knew the dangers, but were not so well acquainted with the remedy for the evil.

LORD BALFOUR OF BURLEIGH, who warmly approved the work of the Association, explained that he was present that afternoon in his private capacity, not as Secretary of State for Scotland. To illustrate this, he told an anecdote of an Indian Civil Servant. On the birth of his son his dependents asked whether they might have a holiday in honour of the event. This put him in great perplexity, and he consulted his clerk, who after serious deliberation gave his opinion. The child, he said, was born to his father in his private and unofficial capacity; therefore the request ought not to be granted. He supported the Bill because it would be a dereliction of duty not to do so; he had not, however, a very profound sense of the utility of deputations; and he did not think that the greatest service that could be done to a measure was its adoption by the Government. The cause of the Registration of Midwives justified continual agitation. The Association had greatly extended its influence, and if it went on on its present lines it need not fear public discussion. The cause was that of the great majority of the wives of the working population; and though there were many women making an honest living as midwives, the abuses were serious and far-reaching. The prejudice against registration was not merely selfish, it was also foolish. It was a sad fact that 72 or 73 per cent. of the blind children who passed through the hands of the skilled oculists at the College for the Blind need not have been totally blind if proper care had been taken at their birth. It was a fact that might well be brought into prominence. "Go on," said Lord Balfour, "as you are going, and I think success is with you; and if opportunity offers of giving this measure a lift from inside, you may count upon me."

Mr. CROMBIE, M.P., urged the Association to appeal to the Parliamentary constituencies and to rouse their interest in the Bill.

Dr. CULLINGWORTH moved "That, considering the large number of persons in practice as midwives, and the danger and suffering to which women and infant children are exposed by any want of knowledge or skill on their part, this Association respectfully urges upon His Majesty's Government the pressing need of legislation in this, as in other countries, to insure their training and supervision in the future," and it was carried.

### A Nurse's Claim for Damages.

AN action was brought last week in the Sunderland County Court by Miss Selina Hawkins Foote, a nurse, against Dr. George Blacker Morgan, as representing the Sunderland Nursing Institute, of which he is chairman, for £47 5s. 6d. as damages. The claim was made up as follows:—Hire of rooms during sickness for six weeks, £9; nursing fee for three weeks, £5 12s. 6d.; loss of salary during sickness, £4 7s. 6d.; chemist's bill, £1 5s. 6d.; extra nourishment, £2; and pain, suffering, and injury to bone, £25. Mr. Meynell appeared for the plaintiff, and Mr. Gawan Taylor for the defendant. Mr. Meynell stated that on January 19th, 1900, plaintiff entered into an agreement with Dr. Morgan on behalf of the Nursing Institute, by which plaintiff agreed to serve as a certificated sick nurse, at a certain salary, and upon certain terms. She undertook to obey the rules of the society and the matron of the institute. One clause stipulated that travelling expenses would be allowed by the society as well as medical attendance. Another rule provided for three weeks' holiday. In October, plaintiff was in bad health, and suffered from an abscess on her right jaw. Dr. Maling, the honorary medical officer of the institution, attended her. The abscess was operated upon, and the wound healed. Plaintiff then returned to work, though she was in poor health. Under the circumstances, she decided to consult her own doctor — Dr. Squance — although the institute desired nurses to be attended by Dr. Maling. The matron had informed the nurses, however, that they could consult their own doctors if they thought fit, but if they did so they would not be allowed to be laid up in the institute, or would a single day off be granted on the ground of ill-health. Miss Foote knew this, but Dr. Squance advised her to stop working. She could not do so at that time, but later on she asked the matron for three days' holiday. It was granted, but directly the matron got to know that plaintiff was going to have her jaw attended to, leave was stopped, although plaintiff had had no holiday during the year. At that time plaintiff was entirely unfit for work, and on the 14th of December she sent in her resignation. She worked out her notice, and the abscess was then re-opened by Dr. Squance, who found that the bone had been injured. Plaintiff suffered a great deal of pain, and could not work for six weeks, entirely owing to the fact that she had not been allowed to have the abscess attended to at the time when it was absolutely necessary.

Plaintiff was then called, and gave evidence bearing out Mr. Meynell's statement.

Cross-examined by Mr. Taylor: She went to Dr. Squance because she wanted further advice. Her reading of the rule was that she could consult another doctor if she wished.

Miss Isabel Wilson, formerly a nurse at the institute, stated that it was customary to communicate with Dr. Morgan about matters that arose.

Dr. Squance spoke to attending the plaintiff. The operation was performed on January 16th, and in his opinion it ought to have been done much earlier. The three days would have been sufficient if she had got them when she asked for them, namely, in the early part of December. He would have been very glad to confer with Dr. Maling, but the nurse objected.

Mr. Taylor submitted that there had been no breach of contract. The plaintiff had admitted that Dr. Maling was diligent and competent.

Mrs. Marriner, the matron, said that she withdrew the leave on learning that the plaintiff was going to be medically attended outside the institute.

Arguments having been adduced by both sides, Judge O'Connor held that the withdrawal of leave was an attempt to import into the terms of the contract a new element therefore the plaintiff was entitled to judgment. Her claims for damages were, however, excessive, and he fixed the amount at 5s. Costs would follow the event. His Honour added that he did not think anything had been said or suggested against the institute or Dr. Maling.

### "The Hospital" Convalescent Fund.

THE Hon. Secretary begs to acknowledge, with many thanks, the receipt of 10s. from Nurse Cecil.

### Examination Questions for Nurses.

TREATMENT BY IRRIGATION.

RESULT OF JUNE COMPETITION.

THE question was as follows:—"How should you arrange treatment to a leg by constant irrigation, if in an outlying district where the proper appliances were not to be had?"

THE FIRST PRIZE.

"Makeshift" is the most successful candidate this month, though her arrangement as to simply placing the lotion bowl on the seat of the chair over the leg is open to some objections, of which she would probably have ample proof in the first hour or so, supposing the patient to be a man; the restlessness and impatience not unfrequently met with in the lords of creation would most certainly move him to plunge the sound leg about, and then—tableau! It is better, if the bowl or can may not be hung from the wall or ceiling, to sling it from the improvised cradle.

THE SECOND PRIZE.

"Perseverance" wins the second prize. Her paper is very good, and would have brought her out first, but that she makes no mention of slinging the limb. It is not well that it should lie soaking night and day in the lotion used for irrigation.

FIRST PRIZE.

Arrange patient in bed with the bad leg nearest the edge and a warm sock on the foot. Then place a piece of mackintosh or American cloth under the leg, arranging a small groove under the sore, with a slight incline over the edge of the bed, to allow the fluid to run into a pail placed underneath for that purpose. Then stand a firm wooden chair on the bed over the leg, with the jug or pail of lotion on it. Take a long strip of flannel, or lamp-wick, or even rope, put it into the lotion, allowing one end to hang over the side to within about two inches of the sore; this will form a syphon, and keep a steady even drip where necessary. If patient is very restless, it is advisable to slightly sling the limb in a broad piece of flannel, using the chair as a cradle, which will give easy movement without displacement.

SECOND PRIZE.

An easy way of applying irrigation to a leg could be accomplished by taking a bottle or jar containing water (usually cold) and suspend above the bed, by means of a staple or nail fixed in the ceiling, a piece of sterilized lamp-wick, a skein of thread, or a few strands of white wool placed half in the water and half out, and made to depend immediately over the part to which it is to be applied. A constant dripping of water is thus supplied to the affected part, which should be covered with a piece of wet lint, or clean white rag that has previously been boiled. Care must be taken of the bed, and a piece of mackintosh if possible to get, if not, a piece of oil baize or American cloth, which the poor usually use as table-covers, will answer the purpose, and should be arranged so as to conduct the water into a suitable receptacle below. The leg must be raised by placing pillows or cushions above and below the injured part. If it is not possible to suspend in any way stand a jug of water on a table or anything that is a little higher than the bed (a round table if it can be had, as it can be pushed over the edge of bed) on the same side as the affected limb; put one end of a long piece of lint, lamp-wick, or thread into the jug, and the other end must be laid on the lint covering the injury; a continuous little stream of water is thus conveyed to the part that requires it.

HONOURABLE MENTION.

"Shamrock," "Nurse Lorne," and "Hygiene" gain honourable mention. A little more care would have given "Nurse Lorne" the First Prize; her answer is really the best; she mentions the good arrangement by which a hole in the bottom of an old can or kettle may be utilised, and also speaks of the simple and obvious plan of putting an old tray or dish under the limb to catch the superfluous lotion, but alas! carelessness is her undoing; after mentioning this practical detail she says "taking care to make a channel by which the dirty lotion may run off!" Why, when there is already a dish? Almost all candidates assume that the irrigation is to be cold. Much more simple if it is so, but it is often ordered to be kept at a stated temperature and that complicates matters.

THE LAST QUESTION TILL THE AUTUMN.

Summer is the idle season and most people take a holiday long or short. The examinations will be resumed in October.

THE EXAMINER.

### Presentations.

CATERHAM ASYLUM.—On Friday night a farewell dance was given to the nursing staff of Caterham Asylum by the retiring medical superintendent, Dr. G. Stanley Elliot. Dancing commenced at 8.30 in the large recreation hall. During an interval Dr. Patrick Campbell (Dr. Elliot's successor) after saying a few appropriate words on behalf of the staff, presented Dr. Elliot with a very handsome writing-desk, revolving chair and book-case, with suitable inscription, as a mark of appreciation, esteem and regard for his many and varied efforts to advance their general welfare and comfort. Dr. Elliot, in responding, expressed his great satisfaction with the staff, and after dwelling feelingly on his past 30 years' association with Caterham Asylum, bade them all an "affectionate farewell." Dancing was again resumed until 11.30, when a very pleasant evening terminated.

PORTSMOUTH PARISH INFIRMARY.—Last week Mr. E. S. Willcocks, assistant dispenser at the Workhouse Infirmary, Portsmouth, was presented by the matron, on behalf of the nursing staff, with a case of cutlery and a teapot, as a recognition of the very kind way in which he had assisted them to prepare for their late examination, and particularly in teaching materia medica and dispensing.

THE WELSH HOSPITAL IN SOUTH AFRICA.—Last week under the presidency of the Mayor of Carnarvon, a meeting was held in that town at which a presentation was made to Miss Lucy Bulkeley-Williams, in recognition of services rendered by her as a member of the nursing staff of the Welsh Hospital in South Africa. It consisted of a handsome gold watch bracelet, set in diamonds, and an address in album form, signed by 130 subscribers. Lady Turner made the presentation, and several short addresses were delivered. Miss Bulkeley-Williams suitably acknowledged the gift and the good wishes expressed.

THE ROYAL ALBERT EDWARD INFIRMARY AND DISPENSARY, WIGAN.—Sister McKerron, the assistant matron, before leaving for her new appointment as matron of Chalmers Hospital, Banff, was presented by the matron, house surgeons, dispensers, and nursing staff of the Royal Albert Edward Infirmary, Wigan, with a pretty Queen Anne silver tea-service, as an expression of their esteem and regard for her, and as an earnest of their sincere wishes for her happiness and success in her new appointment.

### Wants and Workers.

SISTER D. POTTERTON, Burton Villa, Middle Road, Bournemouth, has copies of *The Hospital* since January, 1900, for any nurse who would care to have them and pay the carriage. An immediate reply asked for.

### Where to Go.

THE DOWDESWELL GALLERIES.—An interesting exhibition of pictures by old masters is opening at these galleries this week. The pictures are chiefly from the collection of Monsieur Emil Pacully, of Paris. They originally belonged to the Infante Don Sebastian de Bourbon.

### Echoes from the Outside World.

### AN OPEN LETTER TO A HOSPITAL NURSE.

ENGLAND has of late become such a pageant-loving nation that the public declaration of the coming coronation of Edward VII. has given pleasure to all. The announcement itself was heard by comparatively few persons because there had been no notice that the quaint ceremony was to take place, and such as were present, except the officials, were simply those whose business led them near St. James's Palace, Temple Bar, or the Royal Exchange. The first hint which the public received that anything unusual was about to take place was the appearance of lines of police in the streets wearing their Jubilee medals, the passing of an escort of Guards along Pall Mall, and the hurrying to and fro of carriages. Any endeavour to find out for what purpose the police had assembled was fruitless; they had evidently been bidden not to tell; the only course possible was to stand and wait. Spectators in the vicinity of Marlborough House, had the best opportunity of seeing all that there was to be seen. The King himself, accompanied by the Queen, Princess Victoria, and the three eldest children of the Duke of Cornwall, from a stand erected in the gardens of Marlborough House listened with interest to the declaration that "We have resolved, by the Favour and Blessing of Almighty God, to celebrate the Solemnity of Our Royal Coronation and of the Coronation of Our dearly beloved Consort the Queen upon a day in June next to be hereafter determined, at Our Palace at Westminster." This statement was followed by the announcement of the appointment of, amongst others, "the Most Reverend Father in God Our right trusty and right entirely beloved Councillor Frederick, Archbishop of Canterbury, Primate of All England and Metropolitan." "Our right trusty and right well-beloved Cousin and Councillor Hardinge Stanley, Earl of Halsbury, Our Chancellor of Great Britain," and "Our right trusty and well-beloved Councillor Edward, Lord Ashbourne, Chancellor of that part of Our United Kingdom called Ireland." This proclamation was read from the Friary Court at St. James' Palace. The balcony was draped in purple, and as the clock struck eleven the four trumpeters, dressed in crimson coats heavily embroidered in gold lace, and carrying silver trumpets with banners bearing the Royal Arms attached to them, blew a fanfare. Perhaps they were nervous, or possibly fanfares require much practice to bring them to perfection, but there was a certain want of clearness and precision about the notes which brought a smile to the face of the man and the woman in the street. Next the Norroy King of Arms, his gorgeous costume gleaming in the sunlight, read the declaration in a clear, rich voice, ending with "God Save the King."

---

THE whole ceremony took about a quarter of an hour, then the officials got into carriages and drove to the entrance to the City, where "The Griffin" stands. Here they were confronted by red cords stretched across the street to prevent them passing. More trumpets were blown, the City Marshal advanced to the barrier, inquiring in a loud voice, "Who comes here?" "The Officer at Arms, who demands entrance to the City to proclaim His Royal Majesty's Coronation," was the reply, and the Pursuivant was led to the Lord Mayor, who gave the necessary permission; the cords were removed, and the cavalcade passed on to the Royal Exchange, where a similar ceremony to that held at St. James' Palace took place, and the people afterwards cheered lustily for the King and Queen. It is thought that very probably the 28th of June next year will be the day selected for the Coronation itself, as that is the anniversary of the day upon which Queen Victoria herself received the crown so many years ago.

---

WHETHER we have relatives in the Navy or not, all of us naturally take a keen interest in our sailors, their grievances and their rewards. This feeling has been accentuated of late because of the work done in the relief of Ladysmith by the *Powerful* men, and also by the performance of the sailors on a more recent occasion at Windsor, when, had it not been for the "handy man" stepping into the breach, the solemnity and due order of the funeral procession would have been in serious danger of being destroyed, even had no worse disaster happened. Therefore, the announcement that the King has approved of a Memorial made him by the Lords of the Admiralty praying that some means should exist of recognising valour in the Navy, is a matter for congratulation to us all. Under previous regulations warrant officers or subordinate officers of the Fleet, not holding a commission in the Royal Navy, have been ineligible for appointment to any existing order or decoration. Accordingly, a new decoration, to be designated the "Conspicuous Service Cross," has been instituted. It consists of a silver cross, with one side plain, having on the other in the centre the Imperial and Royal cypher E.R.I., surmounted by the Imperial crown. This decoration will be awarded for meritorious or distinguished services before the enemy, and no person is to be nominated to the Order unless his services shall have been mentioned in despatches by the officer commanding the squadron or force of which he was a member. The award of the decoration is to carry with it the right to append the letters C.S.C. after the holder's name, an honour which will probably be much coveted.

---

IT is, I think, a matter for satisfaction that the three days' motor car race took place from Paris to Berlin, rather than from John o' Groat's to Land's End. First, because in some strange way the fact of French and Germans racing together seems to have done more to create a good understanding between the two nations than any other event since the memorable war of 1870; and secondly, because they were not English children who have been sacrificed to the ungainly monsters who slay so swiftly, and whose mad career it is so impossible to withstand. Those who witnessed the arrival of the successful Frenchman who won the race say that the scene in Berlin was most extraordinary. The machine, long and low in shape, with a cruel-looking prow in front, rushed to the winning post enveloped in a cloud of dust. It was greeted with deafening applause, mingled with cries of alarm from the crowd, who foresaw the possibility of being run into; but M. Fournier pulled up with violent abruptness just two inches from the steward. Inside the car were two strange beings clad in khaki-coloured oilskins, their eyes completely hidden by huge goggles, their faces by dirt and grime. But, undeterred by the formidable appearance of the hero, the only recognisable portion of whom was his yellow moustache, the crowd took him on their shoulders and carried him off triumphantly to the dressing-room, where he ducked his head in a tub and emerged the cleaner in consequence. Then, laden with flowers and wreaths of laurel, dukes and princes congratulated him, and great ladies asked to be presented to him.

---

As to the accidents, much regret is, of course, felt that lives have been lost. In one case it is asserted that, if the motor had not run over the child, it would have charged the crowd, and killed far more victims. As yet there seem to have been only three deaths, but the Austrian count who was thrown out when the car came in contact with a tree is so severely injured that it is uncertain if he can recover. Probably it is the last time that motors will be allowed to race along the public roads anywhere. In Paris an agitation is progressing against the speed they are allowed to attain, forty to fifty miles an hour, and one paper reminds its readers that the great Revolution resulted from aristocrats riding ruthlessly over people. In England hitherto the accidents have generally occurred to the occupants of the car, and a friend of mine warns me against trusting to the perils of a public motor omnibus. She was riding in one—the only inside passenger—the other day, and the floor suddenly became so hot that she felt as if she were being grilled on the top of a kitchen fire. She stopped the vehicle and got outside. She was only just in time, for in a few minutes flames broke out and jugs of water had to be fetched from a private house to extinguish the fire. Of course she continued her journey on foot.

### Everybody's Opinion.

[Correspondence on all subjects is invited, but we cannot in any way be responsible for the opinions expressed by our correspondents. No communication can be entertained if the name and address of the correspondent is not given, as a guarantee of good faith but not necessarily for publication. or unless one side of the paper only is written on.]

### NURSES' READING SOCIETY.

"MISS MOBERLY" writes: I wish to notify to the members of the Nurses' Reading Society my change of address from 24 Portland Place, W., to Langwathly, Belsize Lane, N.W. The falling-off of members is very lamentable, and although I propose to keep on the society for another year, I shall not be able to continue it longer without an increase of members.

### THE NURSES' CO-OPERATION.

"SISTER DORA" writes: It has caused me much anxiety and worry, and much sorrow too, to see the name of the Nurses' Co-operation dragged before the public in the way that it has been lately. I was one of the early members. What happy days those were! How united we were, how we were appreciated, and how rapidly it grew! During the nine years and four months I worked on the staff (and worked hard nearly all the time for numbers of doctors far and wide) I must say I always heard it spoken of in the very highest terms. I always was, and am still, proud to be able to say I was a Co. nurse. I really cannot make out why all this friction has arisen. I hope now that there will be a real settling, and that the Co-operation will recover itself. I am delighted to read that every nurse is up in arms about Miss Hughes sending in her resignation. I am sure she had a real reason. I should also like to say a few words about the Nurses' Home, the De Walden Home. I stayed there often and received the greatest kindness and courtesy. As to being expensive this is all nonsense. Many a time I have had a glass of milk and bread and butter for my supper. No nurse is compelled to pay more than she chooses. Indeed, I was always very happy there. Miss Wells, the matron, was extremely kind to all. The nurses of the Co-operation do not realise how privileged they are to be able to earn such a large sum as they do, and take a rest almost when they like. I think sometimes that we are apt to grow very selfish and dissatisfied with so much liberty. The post filled by Miss Hughes is by no means an enviable one, and it is only by fine tact and splendid management, together with ability and power that she has been so successful. We want a few more matrons like her and a few more loyal nurses.

### THE DUTIES OF ARMY SISTERS.

"A. N. S." writes from Natal: Having read through your number dated April 20th, 1901, I found in an article headed "Eight Months at the Imperial Yeomanry Hospital, Deelfontein," by an Army Nursing Reserve Sister the following statement:—"The regular Army sister is expected to do very little in the wards beyond giving the medicines and stimulants, going round with the doctors and charting the temperatures. She has nothing to do with the distribution of food or bed-making, etc., all work of that kind being done by trained orderlies belonging to the R.A.M. Corps." Now, as an Army sister of thirteen and a half years' standing, I feel that such a statement appearing in print and scattered broadcast needs correction in the same way. An Army sister's duties, under the existing regulations are far more onerous and varied than those of a sister in a civil hospital, and, needless to say, they cannot be readily grasped. She has at once to combine the duties of sister, nurse, probationer, and orderly. She has to teach the raw recruit every detail in nursing—no easy matter with a man. She often has no first-class (trained) orderly, and as there is plenty of heavy work, which is not nursing, left for the junior orderly to do, she must turn her attention to bed-making of helpless patients; in fact, the entire care of these often falls to her lot. Night duty is often done in conjunction with day duty in order that the sister may see an acute case carefully through a crisis; all food, stimulants and medicine being administered by herself, the treatment likewise. Of course, with such cases, an orderly is also on night duty; but he most probably has never seen the case before, therefore no persuasion would induce the sister to leave her case. According to Regulations for Army Medical Services, para. 149, the sister is responsible for the personal cleanliness of patients, also that all medicines, diets and extras are supplied to the patients according to the instructions of the prescribing medical officer. The diets of convalescents are certainly divided into separate portions for each man by the cook in the kitchen before they are sent to the wards, and these again are liable to inspection by the medical officer and sister, but a sick man's diet is entirely given to him under the sister's supervision. For the proper distribution of it during the twenty-four hours, Benton's diet charts are often used. A first-class orderly will readily understand these and can give invaluable help. My experience in the army has been that a sister's patients are ever present with her, whilst in civil hospitals the patients can be handed over to a competent nurse and then the strain is relaxed for a time at least. I am not criticising the style of nursing in the army, to which I belong I am proud to say, but in the interests of army sisters whom I have known and still know, and whose equal in devotion to their patients I have never met in civil life, I wish to disprove such a statement as the one quoted above. Nurses who have never been in the army before the war started in 1899 can in no way be judges of the nursing in the same army in times of peace. Unfortunately, criticisms have been passed and a great many untrue statements have been made, but so far I have heard of no one who has taken up the cudgels, in writing, for the defence of the army nursing service, though I have heard a great deal of indignation expressed in words when so much that is derogatory to them has appeared in print. We are on the eve of some great change, and I hope that some army sisters (regulars) will be asked to give their opinions on the matter, for who can judge better than they where the defects lie?

### Appointments.

GRAMPIAN SANATORIUM, KINGUSSIE, N.B.—Miss I. Balfour-Hope has been appointed nurse-superintendent. She was trained at the Glasgow Western Infirmary, was a private nurse in Edinburgh for over seven years, and has since been matron of a small sanatorium.

GROVE FEVER HOSPITAL.—Miss Florence Ann Cann has been appointed assistant matron. She was trained at Westminster Hospital, and has since been temporary matron at Islington Infirmary, night superintendent at University College Hospital, ward sister at the Hospital for Women, Soho Square, and theatre sister at Wolverhampton General Hospital.

INFECTIOUS DISEASES HOSPITAL, UPNEY.—Miss A. M. Smith has been appointed matron. She was trained at Middlesex Hospital, and has since been nurse at the City of Worcester Isolation Hospital, Enfield Isolation Hospital, and Ilford Isolation Hospital.

METROPOLITAN CONVALESCENT INSTITUTION, WALTON-ON-THAMES.—Miss Florence Frost has been appointed sister. She was trained at the Royal Infirmary, Wigan, and has since been staff nurse at the Westminster Sick Poor Nursing Home, charge nurse at Luke's Hospital, Halifax, and charge nurse at Leeds Beckett Street Infirmary. She has also held an appointment at the Nursing Home, Avenue Thiers, Nice, for the past season.

RHONDDA ISOLATION HOSPITAL.—Miss Edith M. Procter has been appointed matron. She was trained at St. Thomas's Hospital, and has since been senior sister at the Cardiff Sanatorium.

WATFORD UNION INFIRMARY.—Miss Alice Edgcumbe Chevallier has been appointed superintendent nurse. She was trained at Crumpsall Infirmary, Manchester, has since been ward sister at Prescot Union Infirmary and Selly Oak Infirmary, and superintendent nurse at the Atcham Union Infirmary, near Shrewsbury. She holds the L.O.S. certificate.

WREXHAM DISTRICT HOSPITAL.—Miss Clara Ryan has been appointed staff nurse. She was trained at Wrexham Joint Fever Hospital.

YORK COUNTY HOSPITAL.—Miss Helen Topham has been appointed assistant matron. She was trained at the York County Hospital, and the British Lying-in Hospital, Endell Street, London, and has since been sister, for 13 years, at the York County Hospital.

### For Reading to the Sick.

"ALONE, YET NOT ALONE."

THERE is nothing like solitude for teaching us that we are not solitary—nothing like weakness for making us realise what strength may be ours.—*Edna Lyall. In the Golden Days.*

"Alone, yet not alone," so spake
The Saviour in that hour,
When twilight shadows of the cross
Did o'er His pathway lower;
Alone He drained the Passion Cup,
Alone the winepress trod,
"Yet not alone," for One was near,
His Father and His God.

. . . . . . . .

"Alone, yet not alone;" be this
My comfort when I hear
A Voice that summons me to part
From all that made life dear.
"Alone;" no lamp of human love
My pilgrim steps to guide;
"Yet not alone," for Christ will tread
The valley at my side.

*Horatio L. Nicholson, D.D.*

And though
No more thou hear the voice nor feel the hand
Thou lovest, yet thou art not desolate;
I leave thee to a better Friend than I.
Love Him, and trust Him, follow Him with pains—
Not easily—the grace is for the strife;
And whatsoever trial He may lay
Upon thee, trust Him through it, and give thanks;
And when thy heart is heavy, think on Him,
And when thy need is greatest, call on Him.
Hold fast God's promise, and remember this—
Christ will not fail thee.

*Mrs. Hamilton King. The Disciples.*

Why, as we go through life, does one familiar face, and voice, and touch, and step fade away after another? Why are we compelled, as it were, to relax our hold on the things which are seen and temporal, and to live much within the veil? It is because we, as God's children, are being educated up to a world where there were will be one focus for every eye, one object for every affection, one note for every ear, one theme for every tongue, one resting-place for every heart, and one satisfaction for every weary longing—"Jesus only."—*Rev. G. Stopford Ram. Last Words.*

The solitude of life in its ultimate issue is because we were made for a higher companionship. It is just in the innermost sanctuary, shut to every other visitant, that God meets us. We are driven to God by the needs of the heart.—*H. Black.*

Only Christ, who understandeth,
Cometh when the door is fast;
Only He, through moods and manners,
Loveth, loveth to the last.
May the lesson we are learning,
Ever learning, be the same—
"When the doors were shut," by sorrow,
Sin, or failure—"*Jesus came.*"

*S. M. E.*

### Notes and Queries.

**The Editor is always willing to answer in this column, withou any fee, all reasonable questions, as soon as possible.**
**But the following rules must be carefully observed:—**
**1. Every communication must be accompanied by the name and address of the writer.**
**2. The question must always bear upon nursing, directly or indirectly.**
**If an answer is required by letter a fee of half-a-crown must be enclosed with the note containing the inquiry.**

*The Light Treatment of Lupus.*

(127) Will you kindly give me the address of the French manufacturer of the lamp used for the "Light Treatment of Lupus."—*M. H.*

The inventors of the French lamp described in "THE HOSPITAL" NURSING MIRROR of June 22nd, are Messrs. Cortet and Genoud, of Lyons.

*Salary.*

(128) Will you please tell me what salary is due to me on the death of my patient. It was a permanent case, and I entered into the engagement quarterly, salary to be paid same also. I have now to ask the trustees of my patient for it, and wish to make the proper charge.—*A. J.*

If you have been paid quarterly you are certainly entitled to a quarter's salary in lieu of notice.

*"Struma."*

(129) Could you oblige me by giving name and price of a recent book on "struma" which would be of practical use to a nurse working among scrofulus cases in a remote district.—*Sheenie.*

"Struma" and "scrofula" are old-fashioned terms which have been very loosely applied. The doctor in attendance will give directions according to the nature of each case.

*Temperature.*

(130) I have frequently been told that it is a bad sign if a patient's temperature is higher in the morning than at night, yet no one has been able to give me an explanation as to the reason. Can you help me?—*Nurse E. A.*

This is a statement which is often made, and perhaps there is something in it. In normal conditions the temperature tends to rise a little towards evening, and in nearly all fevers this evening rise is much more marked so as to become quite a feature in the case. Now it is a general experience that any marked departure from the normal type in any fever is attended with increased risk, and of course such an alteration of the rhythm of the temperature as is mentioned above would amount to an absolute reversal. Hence one might expect that all was not going on well—that perhaps some complication was arising—and this often turns out to be the case. That is about as far as we can well go.

*Rheumatism.*

(131) Can you oblige by giving me title of a really good book on "Reumatism?" Also price &c.—*Inquirer.*

No, we know no book on "Reumatism." In all books on general medicine there are chapters on Rheumatism, and a good special work on the subject is that by Maclagan.

*Incurable.*

(132) Can you tell me of a home, free or nearly so, for a nurse, fifty-seven years of age, who is suffering from phthisis and heart disease. She is too ill for the hospitals and not ill enough for a home for the dying. She is very cheerful and bright, and the doctor thinks she may live about a year longer. —*M. H.*

St. Peter's Home and Sisterhood, Mortimer Road, Kilburn; and the Home for Consumptive Females, 57 and 58 Gloucester Place, Portman Square; and the Firs Home, Trinity Road, Bournemouth, seem the most likely institutions to benefit the case. The fee for the first is 8s. to 10s.; for the second 7s.; and for the third 10s. 6d. a week.

*Epileptic.*

(133) Can you tell me where an epileptic patient, slightly effected mentally, could be received at between £30 and £40 a year?—*M. H.*

The Meath Home of Comfort for Epileptics, Westbrook, Godalming. Reserved beds £30 a year.

*Capetown.*

(134) 1. Will you kindly tell me what chances trained nurses have in Capetown, or any town in South Africa? 2. Are any nurses wanted there to nurse the plague?—*Liverpool.*

1. You will get the latest returns of nursing prospects in South Africa from the Emigrants' Information Office, 31 Broadway, Westminster, S.W. 2. No. See MIRROR, May 4th: "Plague Nursing in Capetown."

*Advisability.*

(135) May I ask your opinion as to the advisability of the house surgeon going out with members of the nursing staff?—*A Sufferer.*

It is usually considered inadvisable.

*Spinal.*

(136) "Matron" wishes to know if the Editor could kindly tell her of a home for incurables where a girl suffering from spinal disease could be received as paying patient.

The Orthopædic Hospital and Home for Crippled Children, Grove Road, Redland, Bristol. See "Burdett's Hospitals and Charities."

**Standard Books of Reference.**

**"The Nursing Profession: How and Where to Train." 2s. net; post free 2s. 4d.**
**"Burdett's Official Nursing Directory."** 3s. net.; post free, 3s. 4d.
**"Burdett's Hospitals and Charities."** 5s.
**"The Nurses' Dictionary of Medical Terms." 2s.**
**"Burdett's Series of Nursing Text-Books." 1s. each.**
**"A Handbook for Nurses."** (Illustrated.) **5s.**
**"Nursing: Its Theory and Practice." New Edition. 3s. 6d.**
**"Helps in Sickness and to Health." Fifteenth Thousand. 5s.**
**"The Physiological Feeding of Infants." 1s.**
**"The Physiological Nursery Chart." 1s.; post free, 1s. 3d.**
**"Hospital Expenditure: The Commissariat." 2s. 6d.**
**All these are published by THE SCIENTIFIC PRESS, Ltd., and may be obtained through any bookseller or direct from the publishers, 28 and 29 Southampton Street, London, W.C.**

